# Impact of Lesinurad and allopurinol on experimental Hyperuricemia in mice: biochemical, molecular and Immunohistochemical study

**DOI:** 10.1186/s40360-020-0386-7

**Published:** 2020-02-10

**Authors:** Youssef Saeed Alghamdi, Mohamed Mohamed Soliman, Mohamed Abdo Nassan

**Affiliations:** 1 Biology Department, Turabah University College, Taif University, Turabah, 29541 Saudi Arabia; 2 Biochemistry Department, Faculty of Veterinary Medicine, Benha University, Benha, 13736 Egypt; 3 Clinical Laboratory Sciences Department, Turabah University College, Taif University, Turabah, 29541 Saudi Arabia; 4 Pathology Department, Faculty of Veterinary Medicine, Zagazig University, Zagazig, Egypt

**Keywords:** Lesinurad, Ameliorative effects, Hyperuricemia, Gene expression, Kidney affection, XOD activity

## Abstract

**Background:**

Hyperuricemia is an abnormal increase in uric acid levels in the blood. It is the cause of gout that manifested by inflammatory arthritis and painful disable. Therefore, current study evaluated the potential ameliorative impact of Lesinurad and Allopurinol on the kidneys of hyperuricemic mice at the biochemical, molecular and cellular levels.

**Methods:**

Lesinurad and allopurinol alone or in combination were orally administered to hyperuricemic and control mice for seven consecutive days. Levels of uric acid and blood urea nitrogen, along with antioxidants and inflammatory cytokines (IL-1β and TNF-α) were measured in the serum. The mRNA expression of mouse urate anion transporter-1, glucose transporter 9, organic anion transporters, in renal tissues were examined using quantitative real time PCR. Simultaneously, the immunoreactivity of transforming growth factor-beta 1 was examined immunohistochemically.

**Results:**

Lesinurad and allopurinol administration resulted in significant decrease in serum levels of uric acid, blood urea nitrogen, xanthine oxidase activity, catalase, glutathione peroxidase and inflammatory cytokines (IL-1β and TNF-α) reported in hyperuricemic mice. Both partially reversed oxonate-induced alterations in renal mURAT-1, mGLUT-9, mOAT-1 and mOAT-3 expressions, as well as alterations in the immunoreactivity of TGF- β1, resulting in the increase of renal uric acid secretion and excretion. The combined administration of lesinurad and ALP restored all altered parameters in a synergistic manner, improving renal function in the hyperuricemic mouse model employed.

**Conclusion:**

This study confirmed synergistic ameliorative hypouricemic impact of both lesinurad and allopurinol in the treatment of hyperuricemia in mice at the biochemical, molecular and cellular levels.

## Background

Uric acid (UA) is the end product of an exogenous and endogenous pool of purines metabolism. The exogenous pool is dependent on diet especially from animal proteins. The endogenous pool depends on the production of uric acid from the liver, intestines and other tissues [1]. Potential sources of the exogenous pool of hyperuricemia (HU) consists of food (i.e. purine rich products), glucose, and fructose [2], which is controlled by xanthine oxidase, that irreversibly oxidizes xanthine into uric acid (UA) [3, 4]. UA is primarily excreted through the kidneys in urine (65–75%) and to a lesser extent through the gastrointestinal tract (25–35%) [3, 5]. An increase in the level of uric acid in the blood is known as hyperuricemia, and is the cause of gout, which manifests in inflammatory arthritis and painful disabling with acute attacks [6].

HU is defined as an increase in UA levels over 7 mg/dL in men and 6 mg/dL in women [7]. HU is mainly associated with the following: (1) alcohol consumption; (2) a fructose rich diet; (3) excess consumption of seafood or meat; (4) diuretics; (5) some medications; and (6) angiotensin converting enzymes [8, 9]. Gout is associated with precipitation of monosodium urate (MSU) crystals in joints and soft tissues [10, 11]. Deposition of MSU crystals in the big toe, some joints and the ankle are associated with neutrophil infiltration, swelling and pain [12]. The first line of gout treatment is allopurinol (ALP), a xanthine oxidase inhibitor stimulating the renal secretion and excretion of UA [11]. Other anti-inflammatory non-steroidal drugs known to inhibit cyclooxygenase activity (i.e. cortisol, indomethacin and glucocorticoids) are beneficial for the treatment of gout [13]. However, these medications have severe side effects and interactions capable of harming human health [14].

A promising approach for the treatment of HU and its associated complications consists of alternative therapies, i.e. dietary flavonoids and hypouricemic curative agents with suboptimal doses devoid of undesirable ALP side effects. The need to identify safe drugs is therefore vital for both physicians and patients. A new medication used for treatment of HU is lesinurad, commonly named Zurampic (ZUR). ZUR was approved in December 2015 by the US Food and Drug Administration and works on the urate-anion exchanger transporter (URAT1) in combination with ALP [15]. Furthermore, URAT1 is a trans-membrane protein that acts as a urate-specific and organic anion exchanger, being localized in the luminal membrane of the proximal convoluted tubules [16]. URAT-1 action increased urate filtration and reabsorption from proximal convoluted tubules [17]. Lesinurad inhibits URAT1 and Organic Anion Transporters (OATs) controlling uric acid reabsorption, and increases the urinary excretion of uric acid [18]. A previous study postulate that the declined levels of serum uric acid levels takes place in response to the effect of lesinurad on renal urate. The current study therefore examined the ameliorative synergistic impact of lesinurad and ALP on oxonate-induced HU in mice at the biochemical, molecular and histopathological levels.

## Methods

### Chemicals and kits

Potassium Oxonate (PO), agarose, ALP and ethidium bromide were purchased from Sigma-Aldrich (St. Louis, MO, USA). Zurampic was purchased from Ironwood Pharmaceuticals, Inc., Cambridge, MA02142. 100 bp DNA ladder and reverse transcriptase enzymes were from MBI (Fermentas, Thermo Fisher Scientific, USA). Qiazol and Oligo dT were from QIAGEN (Valencia, CA, USA). The kits for catalase, MDA and glutathione peroxidase (GPx) were purchased from Biodiagnostic Co. (Dokki, Giza, Egypt). The kits for glutamate pyruvate transaminase (GPT), glutamate oxalate transaminase (GOT), blood urea nitrogen (BUN) and uric acid were from BIO-MED Diagnostics and EGY- CHEM for lab technology, Badr City, Egypt. Xanthine Oxidase kit (Catalog No: E-BC-K024), mouse IL-1 beta (Catalog No: E-EL-M0037) and mouse TNF-alpha (Catalog NO: E-EL-M0049) were obtained from Elabscience Biotechnology Inc. USA.

### In vivo animals and design

The Scientific Deanship of Taif University, Saudi Arabia, along with its Ethical Committee, approved all procedures used in this study for the project #1–439–6099, based on the NIH Guide for the care and use of laboratory animals. A total of forty-two male Swiss mice, ten weeks old, weighting 30–35 g from college of pharmacy, King Abdel-Aziz University, Jeddah, Saudi Arabia were used for this study. To overcome stress and complete adaptation, the mice were handled manually for 7 days. The animals were kept in a 12/12 h day-dark cycle and were given free access to food and water. Seven groups (6 mice /group) were allocated to the following treatments:

Group 1; negative control (CNT), gained free access to food and water. Group 2; positive hyperuricemic group (HU), received potassium oxonate (PO) intraperitoneally (250 mg/kg bw, once a day at 8:00 am). The dosages of PO and timing were determined as stated above [19]. Time of administration was fixed to avoid noctorinal and diurnal changes in biochemical measurements. Group 3 received orally allopurinol (ALP; 5 mg/kg body weight daily 1 h after PO administration) for 7 days [20]. Group 4 was administered orally ZUR in a dose of 80 mg/kg as stated by Wu et al. [21]. For 7 days, groups 5 and 6 were administered PO at 8.00 am, followed by ALP for Group 5 and ZUR for Group 6 1 h later (9: 00 am). Group 7 was administered PO for 7 days, followed by a combination of ALP and ZUR 1 h later. For treatments in groups from 5 to 7, same doses given in group 3 and 4 were used. At the end of the experimental design (2 weeks), the mice inhaled dimethyl ether and were decapitated. Serum was extracted and stored at − 20 °C until biochemical measurements took place. Kidney and liver tissues were soaked in Qiazol for RNA extraction and in Bowan’s solution for histology and immunohistochemistry examination.

### Xanthine oxidase activity

Xanthine oxidase (XOD) catalyzes hypoxanthine, to form xanthine and superoxide anion free radicals, resulting in a purplish red substance in the presence of electronic receptors and a chromogenic agent. XOD activity can be calculated by measuring the OD value of the purplish red substance at 530 nm in serum samples. For liver tissues, following homogenization in normal saline on ice, in a ratio of one liver tissue and nine for normal saline, homogenate is centrifuged for 10 min and supernatant used for assay. The XOD unite for serum values is U/l, and for liver homogenates is U/g protein tissue. The protocol employed was partially modified in accordance with the method of Haidari et al., [22].

### Serum biochemistry, antioxidants, and cytokines assessments

The serum levels of liver and kidney biomarkers were assayed calorimetrically, using specific commercial kits based on the manufacturer’s instruction manual. Antioxidants such as malondialdehyde (MDA), catalase and glutathione peroxidase as well as cytokines were measured spectrophotometrically using commercial ELISA kits explained in chemicals section based on kit attached description manual.

### RNA extraction, cDNA synthesis and quantitative real time PCR (qRT-PCR)

Total RNA was extracted as described before [23] from the kidney tissues. Frozen samples were homogenized in a homogenizer. Chloroform (300 μL) was added to the homogenate, then centrifuged at 4 °C with 12,000 rpm for 10 min. The supernatant was separated and an equal volume of isopropyl alcohol was added then centrifuged at 4 °C with 12,000 rpm for 15 min. The pellets of RNA were dissolved in Diethylpyrocarbonate (DEPC) water after washing with 70% alcohol and air drying. The integrity of RNA was confirmed [24]. 3 μg of extracted RNA and 0.5 ng oligo dT (Qiagen Valencia, CA, USA) was denatured following incubation in a Bio-Rad T100™ Thermal Cycler at 70 °C for 5 min. Denatured RNA was reverse transcribed after the addition of 2 μL of 10 mM dNTPs, 100 U of M-MuLV (SibEnzyme, Ak, Novosibirsk, Russia) and 2 μL of 10X RT-buffer, before being incubated in a Bio-Rad T100™ Thermal Cycler for 1 h at 37 °C, then for 10 min at 90 °C, to ensure enzyme inactivation. For quantitative real time PCR analysis (qRT-PCR), primers for the examined genes (Table 1) were designed using GenScript Real-time PCR (TaqMan) Primer Design (https://www.genscript.com/tools/real-time-pcr-taqman-primer-design-tool). Each PCR reaction consisted of 1.5 μl of 1 μg/μl cDNA, 10 μl SYBR Green PCR Master Mix (Quanti Tect SYBR Green PCR Kit, Qiagen, Valencia, CA, USA), along with 1 μM of forward and reverse primer for each examined gene and nuclease free H_2_O to a final volume of 20 μl. Reactions were run and analyzed in Applied Biosystem 7500 Fast Real time PCR Detection system. qRT-PCR conditions are: 95 °C for 10 min (first denaturation) and forty cycles of 95 °C for fifteen seconds (second denaturation stage) followed by 60 °C for 1 min (annealing and extension stage). The critical threshold (Ct) of the target gene was normalized with quantities (Ct) of the housekeeping gene (β-actin), using the formula x = ^2 − ΔΔ^Ct, where there is x = fold difference relative to the control.

**Table 1:** The primers used for quantitative real time PCR (qRT-PCR)

Gene	Product size (bp)	Accession number	Direction	Sequence (5′-3′)
mOAT-1	183	NM_008766.3	Sense	GACAGGGTCTCATCCCTAGC
			Antisense	GTCCCTGACACACTGACTGA
mOAT-3	153	NM_001164635.1	Sense	TACAGTTGTCCGTGTCTGCT
			Antisense	CTTCCTCCTTCTTGCCGTTG
mURAT-1	145	NM_009203.3	Sense	GATAGGTTTGGGCGCAGAAG
			Antisense	TCATCATGACACCTGCCACT
mGlut-9	153	NM_001102415.1	Sense	TTCGGGTCCTTCCTTCCTCTA
			Antisense	GGACACAGTCACAGACCAGA
mβ-actin	143	Nm_007393.5	Sense	CCAGCCTTCCTTCTTGGGTA
			Antisense	CAATGCCTGGGTACATGGTG

### Histological and immunohistochemistry analyses of kidney

The kidney tissue was dehydrated and embedded in paraffin, then sectioned at 5 μm. The slides were subsequently stained with hematoxylin and eosin (H&E) and the morphological changes were examined using a microscope (Eclipse 80i, Nikon, Japan), with images being captured by a digital camera (Fuij Co., Sapporo, Japan).

For immunohistochemistry, the paraffin-embedded renal sections were deparaffinized, rehydrated and immersed in H2O2 (3%) for 10 min, in order to block any peroxidase activity. Following this, the slides were washed in phosphate buffer saline. Nonspecific binding sites were blocked by bovine serum albumin (5%) prior to the addition of TGF-β1 polyclonal antibody in a dilution of 1:300 overnight at 4 ^°^C. The slides were then washed in PBS and incubated with a secondary antibody, developed with 3.3′-diaminobezidine tetrahydrochloride and counterstained with hematoxyline.

### Statistical analysis

Data are means ± standard error of six values collected from six different mice per each treatment. Data were analyzed using one-way ANOVA (analysis of variance) setting the probability level *P* < 0.05, with the individual comparisons obtained by Duncan’s multiple range tests for SPSS software version 11.5 for Windows (SPSS, IBM, Chicago, IL, USA). The probability level *P* < 0.05 was considered statistically significant.

## Results

### The impact of Lesinurad and ALP on liver and kidney biomarkers in hyperuricemic mice

**Fig. 1:**
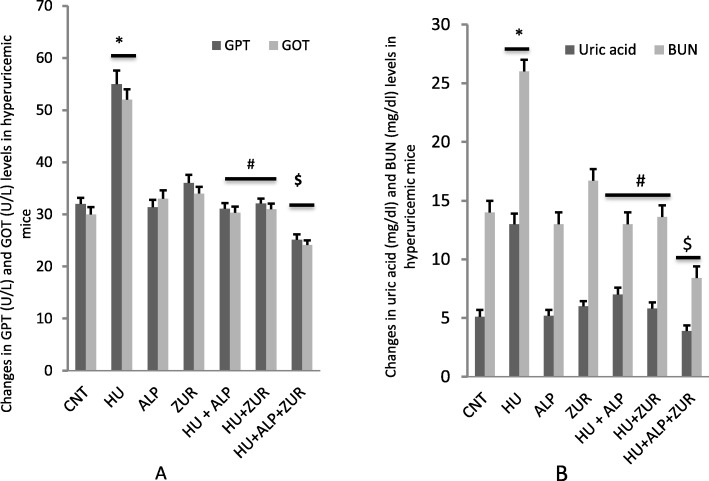
The impact of ZUR on changes in GPT, GOT, uric acid and BUN in hyperuricemic mice. Data are presented as means ± *SE* of six different mice. *p < .05 vs control group; #*p* < .05 vs HUR group and $*p* < .05 vis either HU+ ALP or HU + ZUR groups. CNT: control; HU: hyperuricemia; ALP; ZUR. Units of GPT and GOT are U/L, and for uric acid and BUN are mg/dl

Hyperuricemic group showed an increase in serum levels of GPT, GOT, uric acid and BUN. HU group received either ALP or ZUR showed a decrease in GPT, GOT, uric acid and BUN levels (Fig. 1a-b). Co-administration of ALP and ZUR revealed an ameliorative and additive synergistic effect (P < 0.05) on the normalization of GPT, GOT, uric acid and BUN levels (Fig. 1a). It should be noted that ZUR revealed same effect induced by ALP in hyperuricemic administered mice.

### The impact of Lesinurad and ALP on serum and hepatic XOD activity

**Fig. 2:**
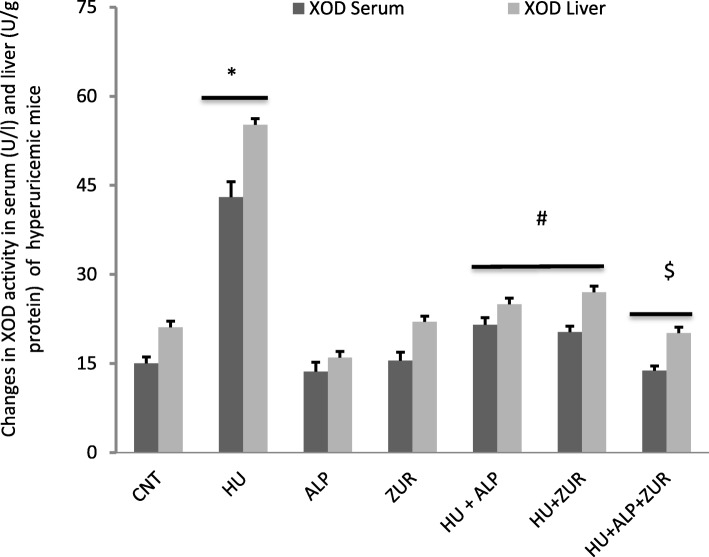
The impact of ZUR on changes in XOD activity in the serum and liver of hyperuricemic mice. Data are presented as means ± *SE* of five different mice. *p < .05 vs control group; #*p* < .05 vs HUR group and $*p* < .05 vis either HU+ ALP or HU + ZUR groups. XOD: xanthine oxidase; CNT: control; HU: hyperuricemia; ALP; ZUR. Measured unites for serum activity of XOD is U/l while for liver tissues is U/ g protein

As shown in Fig. 2, there was an increase of XOD activities in serum and liver of hyperuricemic mice., This increase in XOD activity were significantly normalized to control levels in ALP and ZUR administered hyperuricemic mice. Combination treatment, using both ZUR and ALP, induced an additive and synergistic decrease in XOD activity compared to both hyperuricemic ALP and hyperuricemic ZUR treated groups.

### The impact of Lesinurad and ALP on antioxidant activities altered by hyperuricemia

**Fig. 3:**
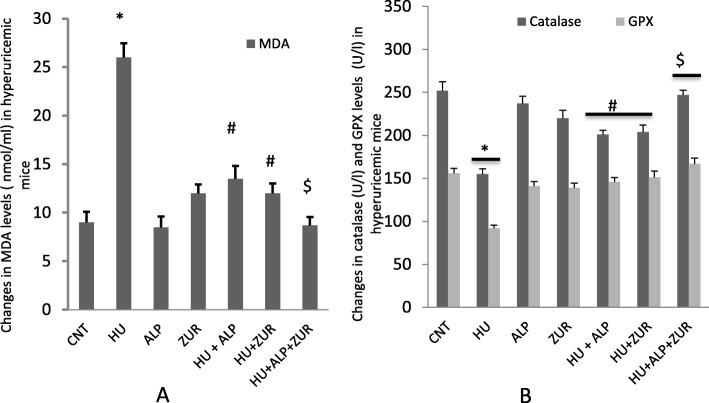
The impact of ZUR on changes in MDA, catalase and GPx in hyperuricemic mice. Data are presented as means ± *SE* of five different mice. *p < .05 vs control group; #*p* < .05 vs HUR group and $*p* < .05 vis either HU+ ALP or HU + ZUR groups. MDA: malondialdehyde; GPx: glutathione peroxidase; CNT: control; HU: hyperuricemia; ALP; ZUR. Units of MDA is nmol/ml, while for catalase and GPx are U/l.

HU increased tissue degradation, represented by an increase in levels of MDA (Fig. 3a), which was normalized in hyperuricemic mice by both ALP and ZUR treatment. HU decreased catalase and GPx levels that were readjusted following ALP administration, and to a lesser extent for ZUR administered groups (Fig. 3b). Administration of ZUR to hyperuricemic mice, together with ALP, induced an additive ameliorative effect on the changes induced on MDA, catalase and GPx levels (Fig. 3a, b).

### The impact of Lesinurad and ALP on changes in cytokines altered in hyperuricemic mice

**Fig. 4:**
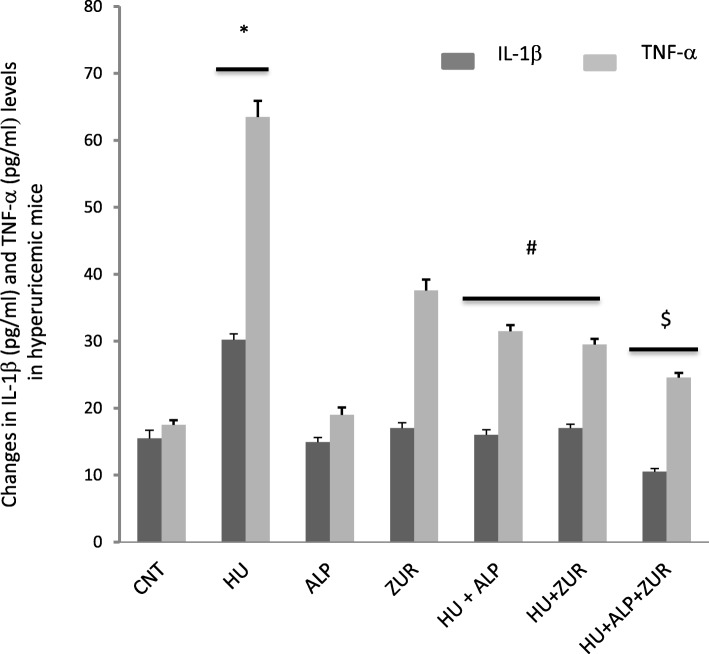
The impact of ZUR on changes in IL-1b and TNF-a in hyperuricemic mice. Data are presented as means ± *SE* of five different mice. *p < .05 vs control group; #*p* < .05 vs HUR group and $*p* < .05 vis either HU+ ALP or HU + ZUR groups. IL-1b: interleukin-1 beta; TNF-a: tumor necrosis factor alpha; CNT: control; HU: hyperuricemia; ALP: l; ZUR. Measured units of IL-1 and TNF are pg/ml

Figure 4 demonstrates the changes in serum levels of IL-1β and TNF-α. HU induced a state of inflammation, with a significant increase in serum levels of IL-1β and TNF-α (*P* < 0.05). Administration of ALP and ZUR to hyperuricemic mice normalized both IL-1β and TNF-α levels. Co-administration of ALP and ZUR induced a clear synergistic inhibitory effect on IL-1β and TNF-α ((*P* < 0.05, Fig. 4).

### The impact of Lesinurad and ALP on mRNA expression of renal genes associated with hyperuricemia

**Fig. 5:**
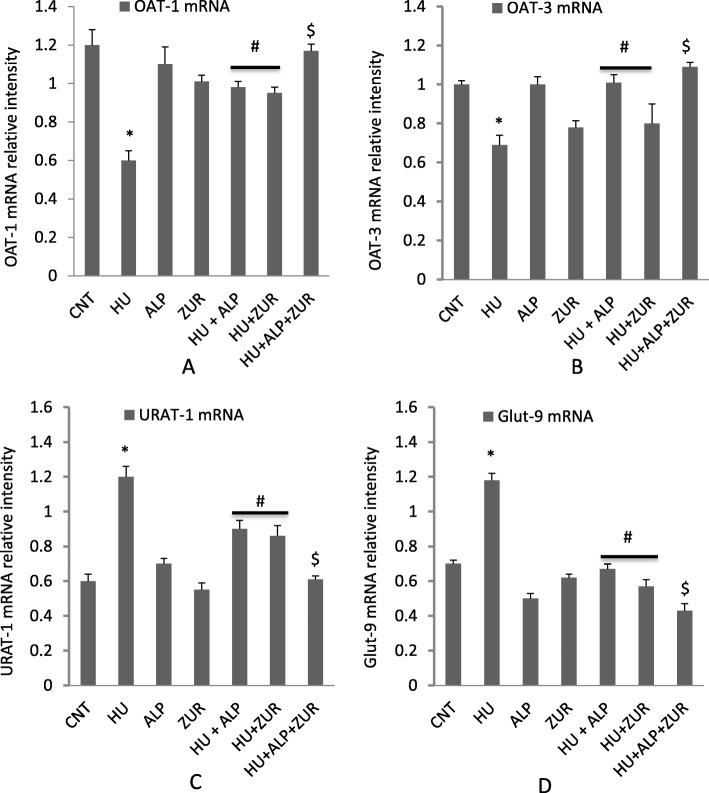
The ameliorative impact of ZUR on mRNA expression of OAT-1, OAT-3, URAT-1 and GLUT-9 in hyperuricemic mice by real time PCR. Graphic presentation of renal mRNA levels by real-time PCR analysis of OAT1 (**a**), OAT3 (**b**), URAT-1(**c**) and GLUT-9 (**d**) in different groups of mice after normalization with beta actin. *p < .05 vs control group; #*p* < .05 vs HUR group and $*p* < .05 vs either HU+ ALP or HU + ZUR groups

This current study examined the expression levels of genes responsible for urate excretion and reabsorption in the kidneys (mOAT-1, mOAT-3, mURTA-1 and mGLUT9). As shown in Fig. 5, in comparison to the mice in the control group, oxonate administration induced a significant down-regulation of mRNA expression of mOAT-1 and mOAT-3 in mice kidneys, alongside a significant up-regulation of the mURAT-1 and mGlut-9 expressions (*p* < 0.05). The alteration in the mRNA expression of urate transporter-related genes was consistent with the elevation of serum uric acid and BUN levels. ALP and ZUR treatment alone showed a significant down-regulation in mURAT-1 and mGlut-9 mRNA levels, as well as up-regulation in mOAT-1 and mOAT-3 expression (Fig. 5). The additive synergistic effect on altered genes could be clearly observed when ALP and ZUR were co-administered to the hyperuricemic group.

### The impact of Lesinurad and allopurinol on renal histology and TGF-β1 immunoreactivity in hyperuricemic mice

**Fig. 6:**
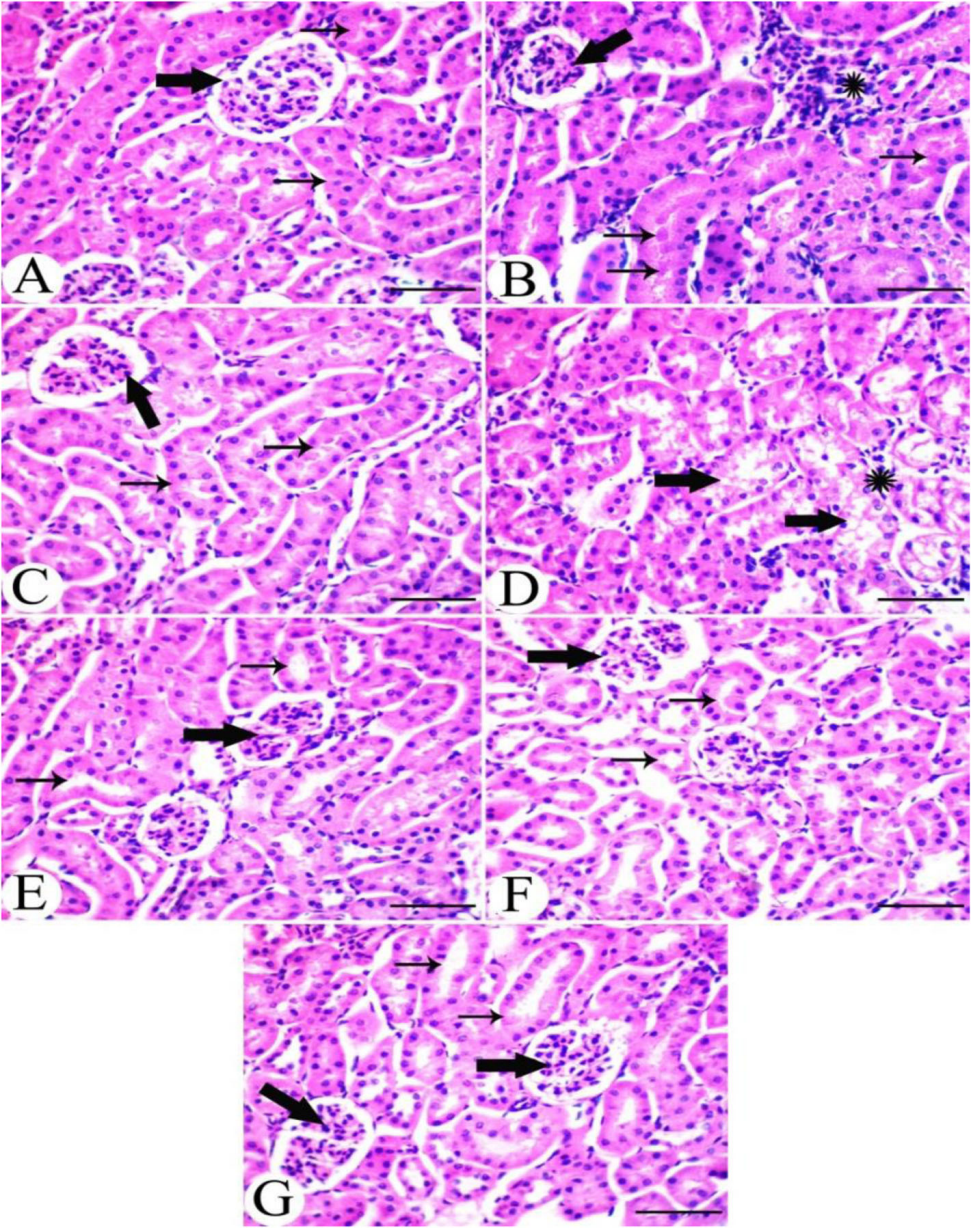
Histopathological examination of Kidneys. **a**. A kidney from the control group, showing a normal histological picture with normal glomerular (thick arrow) and tubular (thin arrows) architecture. **b**. A kidney from the hyperuricemic group, showing shrinkage of glomerular tufts (thick arrow) with periglomrular and interstitial (*) round cells infiltration. Tubular lumina showing obvious urate crystals occluding the lumina (thin arrows). **c**. A kidney from the ALP group, showing no marked change in renal histology with normal glomerular (thick arrow) and tubular (thin arrows) architecture. **d**. A kidney from the ZUR administered group, showing degeneration of renal tubules (thick arrows) with few interstitial round cells infiltration (*). **e**. A kidney from the hyperuricemic group, treated with ALP, showing restoration of normal glomerular (thick arrow) and tubular (thin arrows) architecture. **f**. A kidney from the hyperuricemic group, treated with ZUR alone, showing a slight restoration of normal glomerular (thick arrow) and tubular (thin arrows) picture with the presence of interstitial oedema. **g**. A kidney from the hyperuricemic group, treated with ZUR and ALP, showing a normal histological picture of both glomerular (thick arrow) and tubular (thick arrow) tissue, with the absence of urate crystals. H&E. Scale bar = 50 μm

Histopathological examination revealed that the kidneys of the control group demonstrated a normal histological picture, including normal glomerular and tubular architecture (Fig. 6a). However, the kidneys of the hyperuricemic group revealed shrinkage of glomerular tufts with periglomrular and interstitial round cells infiltration. Tubular lumina showed obvious urate crystals occluding the lumina (Fig. 6b). The kidneys of the ALP administered group demonstrated no marked change in renal histology (Fig. 6c), while the kidneys of the ZUR administered group revealed degeneration of renal tubules with a few interstitial round cells infiltration (Fig. 6d). The kidneys of the hyperuricemic group treated with ALP showed restoration of normal glomerular and tubular architecture (Fig. 6e), while those administered only with ZUR demonstrated a slight restoration of a normal picture, with the presence of interstitial oedema (Fig. 6f). The kidneys of the hyperuricemic group treated with both ZUR and ALP demonstrated a normal histological picture of both glomerular and tubular tissue, including an absence of urate crystals (Fig. 6g).

**Fig. 7:**
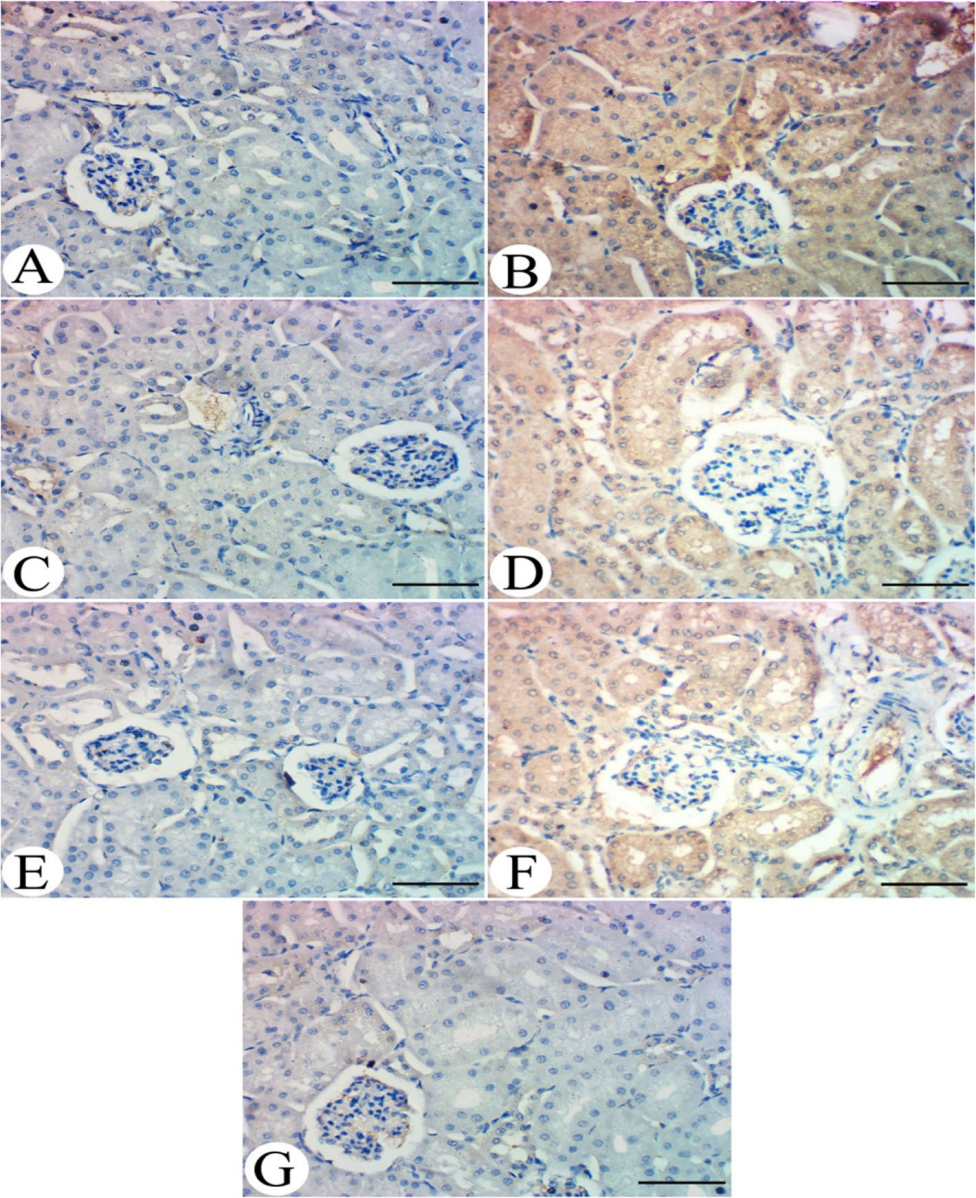
Immunohistochemical examination of TGF-β1. **a**. A kidney from the control group showing no expression of TGF-β in renal tissues. **b**. A kidney from the hyperuricemic group, showing a prominent expression of TGF-β1 in renal tubular tissue. **c**. A kidney from the ALP group, showing no marked expression of TGF-β1 in renal tissue. **d**. A kidney from the ZUR administered group, showing prominent expression of TGF-β1 in renal tubular tissue with moderate intensity. **e**. A kidney from the hyperuricemic group, treated with ALP shed absence of TGF-β1 immunoreactivity in tubular tissue. **f**. A kidney from the hyperuricemic group, treated with ZUR alone, showing a prominent moderate intensity of TGF-β1 in renal tissue. **g**. A kidney from the hyperuricemic group, treated with ZUR and ALP, showing glomerular and tubular tissue with no TGF-β1 expression. H&E. Scale bar = 50 μm

Immunohistochemical examination of kidney for TGF-β1 immunoreactivity revealed that the kidneys of hyperuricemic group showed a clear and strong expression for TGF-β1 in kidney tissues (Fig. 7b) in comparison to control group which showed no immunoreactivity (Fig. 7a). Same is reported for ALP administered group which showed no marked expression of TGF-β1 in renal tissue (Fig. 7c). The kidneys of the ZUR group revealed a very faint expression of TGF-β1 in renal tubular tissue, having moderate intensity (Fig. 7d). In addition, the kidneys of hyperuricemic group treated with ALP demonstrated an absence of TGF-β1 expression in tubular tissues (Fig. 7e). The kidneys of hyperuricemic group treated with ZUR alone showed a moderate intensity for TGF-β1 in kidney tissues (Fig. 7f). However, the kidneys of the hyperuricemic group treated with ZUR and ALP showed glomerular and tubular tissue lacking of any TGF-β1 immunoreactivity (Fig. 7g).

## Discussion

As noted above, hyperuricemia is a cause of gout, as well as a number of clinical disorders, including: chronic kidney disease; hypertension; diabetes; cardiovascular disorders; dyslipidemia; endothelial dysfunction; and atherosclerosis. HU is associated with an increase in the production of oxygen free radicals, oxidative stress and up-regulation of pro-inflammatory cytokines and mediators [13, 25]. The management of gout, cardiovascular and metabolic disorders depends on the activity of xanthine oxidase. The key for hyperuricemia control includes the inhibition of an overproduction of uric acid, along with inflammation and oxidative stress [26].

As previously discussed, uric acid induces a state of inflammation in the kidneys and causes an inflammatory reaction. This accords with a previous study reported relatively similar findings when employing Nuciferine [27]. A number of researchers have confirmed that gout shares many pathogenetic features associated with other inflammatory disorders, i.e. a rapid increase in the secretion of some pro-inflammatory cytokines (IL-1β, IL-6 and TNF-α) [28, 29]. The current study identified that the use of ALP or ZUR alone failed to reduce levels of IL-1β and TNF-α in hyperuricemic mice, suggesting that the co-administration of ALP and ZUR exerted their anti-inflammatory effect to prevent development of gout from HU.

As known, catalases and peroxidases are oxidoreductases that are involved in the molecular defensive mechanisms against reactive oxygen species to counter act the harmful effect of H_2_O_2_ [30]. The increased oxidative stress that occurs with cell damage and inflammation in mice is alleviated by an increase in catalase expression and or secretion [31]. In the parallel, the antioxidant enzyme; glutathione peroxidase (GPx); helps in scavenging cellular free radicals. GPx prevents lipid peroxidation and maintain intracellular homeostasis [32]. In current study, hyperuricemia increased ROS due to the increase in uric acid levels [33]. A significant decline was observed in the serum levels of catalase and GPx activities, accompanied by a significant increase in MDA levels in the hyperuricemic group compared to control. ALP (either alone, or in combination with ZUR) significantly normalized, and reversed, changes in the measured serum levels of antioxidants and the lipid peroxidation marker in hyperuricemic mice, thus suggesting that ALP and ZUR increased antioxidant enzyme activities through their impact on oxidative stress biomarkers.

Xanthine oxidase inhibitors (ALP) are used as first-line therapy for patients with chronic gout, due to factors including availability, efficacy and low cost. However, ALP fails to lower the serum urate to the target level in a substantial subset of adherent patients. This results in the advice for Lesinurad therapy to be taken together with ALP. Lesinurad is a novel selective uricosuric, capable of overcoming the above limitations, while also proving effective in patients having an inadequate response to ALP monotherapy.

Efforts have been made, over a number of decades, to find a wide range of sufficient and safe urate lowering drugs. The close association between HU, metabolic and cardiovascular comorbidities has raised further interest in the development of novel urate-lowering drugs [34]. Uricosurics remain the second choice for treatment of HU and gout, with all recent prescriptions supporting the combination of uricosurics and ALP once monotherapy of each has proved ineffective [35].

Lesinurad is a selective URAT-1 inhibitor approved for HU treatment associated with gout, in combination with ALP. Its exact molecular mechanism is not fully elucidated in animal models. In clinical trials using healthy volunteers, a single dose of ZUR significantly reduced serum UA [35–37], with its efficiency being due to approximately a third of the drug being excreted from the kidneys [36]. The drug should be taken for patients who are refractory to ALP therapy [38]. These current results prompted an investigation into the beneficial action of ZUR on genes capable of validating the excretion and secretion of uric acid [39].

Transporters play important roles in the pharmacology of xenobiotics that start with the recognition of the key contribution of P-glycoprotein to drug properties including biliary excretion, intestinal absorption, penetration the blood-brain barrier and drug-drug interactions [40]. Over 400 transporters expressed in various tissues throughout the body, comprising members of the solute carrier ATP binding cassette protein families have been reported [40]. OATs family comprises a group of over 10 trans-membrane proteins [41]. OAT1 to − 5 are expressed mainly in the kidney, other members are expressed in remaining other tissues. OATs proteins act to maintain kidney homeostasis as urate efflux transporters [41]. Lesinurad has been reported to regulate OAT-1 and OAT-3 expression in in vitro studies [42]. That means, lesinurad and ALP interacts on the kidney to facilitate and increase urate excretion. The current study confirmed that lesinurad has the potential to act in synergistic way to control the expression of URAT-1, OAT 1 and − 3 in kidney tissues to increase urate excretion and secretion.

Serum BUN levels form the markers of renal dysfunction. In addition, mURAT-1 is the main regulator for urate reabsorption (50%), playing a key role in the homeostasis of urate [16]. The glucose transporter 9 (GLUT9) is a protein responsible for urate reabsorption [43], while OAT-1 and OAT-3 are responsible for renal primary urate excretion [44]. This suggests that abnormalities in renal urate transporters may have important implications for the impairment of uric acid excretion, along with HU. The findings of the current study indicate that HU up-regulated mURAT-1 and mGLUT-9 and down-regulated mOAT-1 and mOAT-3, while co-administration of Lesinurad and ALP induced ameliorative synergistic effects. In addition, ZUR and ALP induced up-regulation in mOAT1 and mOAT-3 mRNA, alongside down-regulation in mURAT1 and mGLUT9 in the kidneys of hyperuricemic mice, thus indicating an enhancement of urate excretion reducing serum UA levels. This study confirm that ZUR demonstrates additional uricosuric effects in the presence of ALP, which are mediated through renal mOAT1, mOAT-3, mURAT1 and mGLUT9 regulation in hyperuricemic mice.

HU results in fibrosis and renal tissues injuries involving inflammation and fibroblast expansion with high levels of sodium in extracellular fluids due to an increase in uric acid levels resulting in tissue nucleation [45], thus leading to inflammation influencing the biology of renal interstitial cells [45]. The increase in UA causes the expansion of fibroblasts, as well as up-regulation in the immunoreactivity of profibrotic factors (TGF-β1), confirming the activation of fibrotic pathways in hyperuricemic patients [46]. As demonstrated in Fig. 7, this alteration in TGF-β1 was confirmed during HU and normalization following co-administration of ZUR and ALP.

## Conclusions

**Fig. 8:**
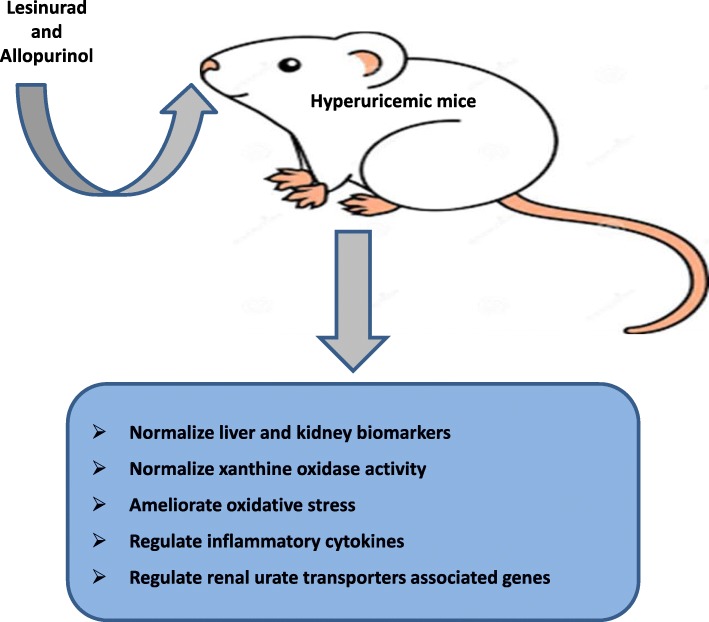
Schematic illustration for the ameliorative effects of lesinurad and allopurinol on hyperuricemia

The present study confirmed that ALP and ZUR co-administration being capable of lowering serum UA. In addition, they led to an additive synergistic decrease in XOD activity in serum and liver tissues and ameliorated induced oxidative stress and changes in pro-inflammatory cytokines. Furthermore, ALP and ZUR co-administration synergistically down-regulated the mRNA expression of URAT1 and GLUT9, and up-regulated the mRNA expression of OAT1 and OAT-3 in hyperuricemic mice. Furthermore, both ALP and ZUR acted together to improve kidney pathomorphology. All ZUR and ALP effects are summarized in Fig. 8. This study therefore suggests the advantages of the co-administration of ALP and ZUR for HU therapy for their beneficial effects on kidney at the biochemical, molecular and cellular levels.

## Data Availability

Data are available up on request.

## Abbreviations

ALP: Allopurinol
BUN: Blood urea nitrogen
CNT: Control
CT: Cycle threshold
DEPC: Diethylpyrocarbonate
GOT: Glutamate oxalacetate transaminase
GPT: Glutamate pyruvate transaminase
GPx: Glutathione peroxidase
H and E: Hematoxylin and eosin
HU: Hyperuricemia
IL-1β: Interleukin-1 beta
MDA: Malondialdehyde
mGLUT9: Mouse glucose transporter 9
M-Mul V: Moloney Murine Leukemia Virus
MSU: Monosodium urate
mURAT1: Mouse urate anion transporter 1
NF_k_B: Nuclear factor kappa-b
OATs: Organic anion transporters
PBS: Phosphate buffer saline
PO: Potassium oxonate
qRT-PCR: Quantitative real time polymerase chain reaction
RNA: Ribonucleic acids
TBE: Tris-borate-EDTA
TGF-1β: Transforming growth factor-1 beta
TNF-α: Tumor necrosis factor-alpha
UA: Uric acid
XOD: Xanthine Oxidase
ZUR: Zurampic
